# Stochastic Cell Fate and Longevity of Offspring

**DOI:** 10.22074/cellj.2017.3919

**Published:** 2017-08-19

**Authors:** Faezeh Dorri, Hamid Pezeshk, Mehdi Sadeghi

**Affiliations:** 1 Institute of Biochemistry and Biophysics, University of Tehran, Tehran, Iran; 2 School of Mathematics, Statistics and Computer Science, College of Science, University of Tehran, Tehran, Iran; 3 National Institute of Genetic Engineering and Biotechnology, Tehran, Iran; 4 School of Biological Sciences, Institute for Research in Fundamental Sciences (IPM), Tehran, Iran

**Keywords:** Decision-Making, Cell Fate, Stochastic, Mathematical Model, Fitness

## Abstract

**Objective:**

Cellular decision-making is a key process in which cells with similar genetic and environmental background make dissimilar decisions. This stochastic process, which happens in prokaryotic and eukaryotic cells including stem cells, causes cellular diver- sity and phenotypic variation. In addition, fitness predicts and describes changes in the genetic composition of populations throughout the evolutionary history. Fitness may thus be defined as the ability to adapt and produce surviving offspring. Here, we present a mathematical model to predict the fitness of a cell and to address the fundamental issue of phenotypic variation. We study a basic decision-making scenario where a bacteriophage lambda reproduces in *E. coli*, using both the lytic and the lysogenic pathways. In the lytic pathway, the bacteriophage replicates itself within the host bacterium. This fast replication overcrowds and in turn destroys the host bacterium. In the lysogenic pathway, however, the bacteriophage inserts its DNA into the host genome, and is replicated simultaneously with the host genome.

**Materials and Methods:**

In this prospective study, a mathematical predictive model was developed to estimate fitness as an index of survived offspring. We then leverage experi- mental data to validate the predictive power of our proposed model. A mathematical model based on game theory was also generated to elucidate a rationale behind cell decision.

**Results:**

Our findings indicate that a rational decision that is aimed to maximize life expec- tancy of offspring is almost identical to bacteriophage behavior reported based on experi- mental data. The results also showed that stochastic decision on cell fate maximizes the expected number of survived offspring.

**Conclusion:**

We present a mathematical framework for analyzing a basic phenotypic variation problem and explain how bacteriophages maximize offspring longevity based on this model. We also introduce a mathematical benchmark for other investigations of phenotypic variation that exists in eukaryotes including stem cell differentiation.

## Introduction

Prokaryotic and eukaryotic cells make decisions on their cell fate stochastically under similar genetic and environmental conditions (1). A similar pattern occurs in stem cells, where identical cells have different fates. Waddington described the process of stochastic decision-making in stem cells by simulating the circumstances in which a ball rolls down onto a slanted landscape with a bifurcated valley. Although, the bi-stable gene regulatory network quantitatively characterizes this process in some single cells, however, for many cells a quantitative model for describing this process is still unavailable. Such stochastic cell decision-making creates cellular diversity and increases chance of survival in varying environment. Fitness, as a central idea in natural selection, is the ability of an organism to survive and reproduce itself in a competitive environment (2-4). Traits or characteristics of an organism, i.e. phenotypes, determine the fitness of an organism. The interaction of genotypic variation and differing environments may cause this phenotypic variation in organisms. For example, trees with larger leaves, as a heritable phenotype, are selected in darker environments due to their environmental adaptation (5- 7). Nevertheless, in stochastic cell decisionmaking, organisms with similar genotypes that are living in identical environmental conditions display different phenotypes. This phenotypic variation is considered as a risk-reducing strategy in many organisms dealing with environmental variation (8-11).

The natural selection process chooses organisms that fit better to the existing environment and it is these that survive and transmit their genome to the next generations (12, 13). In other words, fitness may be defined as an improvement in survival of an organism (14-17) or by the ability of an organism to produce offspring (18, 19). However, finding an exact mathematical model, as a quantitative measure, for fitness is still controversial. Lysislysogeny decision in bacteriophage lambda is a fundamental decision-making process where bacteriophages with similar genotypes living in an identical environment make different decisions by gaining various phenotypes (20- 22). Bacteriophage lambda as a virus is not able to reproduce by itself and needs a host for its reproduction. After bacteriophage lambda infects the bacterium *E. coli*, a decision should be made between the lytic and the lysogenic pathways. In the lytic pathway, the bacteriophage rapidly replicates itself inside the host bacterium and breaks down the host and releases its content in the environment. On the other hand, in the lysogenic pathway, bacteriophage lambda inserts its genome into the *E. coli* genome and reproduces by *E. coli* reproduction (23-25). Bacteriophages try to optimize their fitness by having to face a trade-off between the lytic and the lysogenic pathways (22, 26, 27). If many bacteria are present in the environment, the lytic pathway is preferred since the released offspring is more likely to find a host during their journey. However, infecting all bacteria make the lytic pathway an inefficient choice since there would not be enough hosts for the released bacteriophages. Hence, a mixture of both lysis and lysogeny is required to optimize the fitness of the bacteriophages.

In our study, we aimed to investigate the lysis-lysogeny decision-making problem in bacteriophage lambda as a basic cellular phenotypic variation problem. In this scenario, bacteriophages have identical genomes and environment but with different decisions. We developed a mathematical model to describe a rationale in the bacteriophage decisionmaking process. Our model gives an exact quantitative measurement of fitness for every possible decision. In particular, we define fitness as the expected number of survived bacteriophage offspring and propose a model to estimate the total number of survivors in the environment. Moreover, we evaluated our model by comparing our expectations with data from real world experiments and show that a rational decision based on our model matches well with the behavior of bacteriophage lambda reported in experimental studies.

### The lysis-lysogeny decision

**Fig.1:**
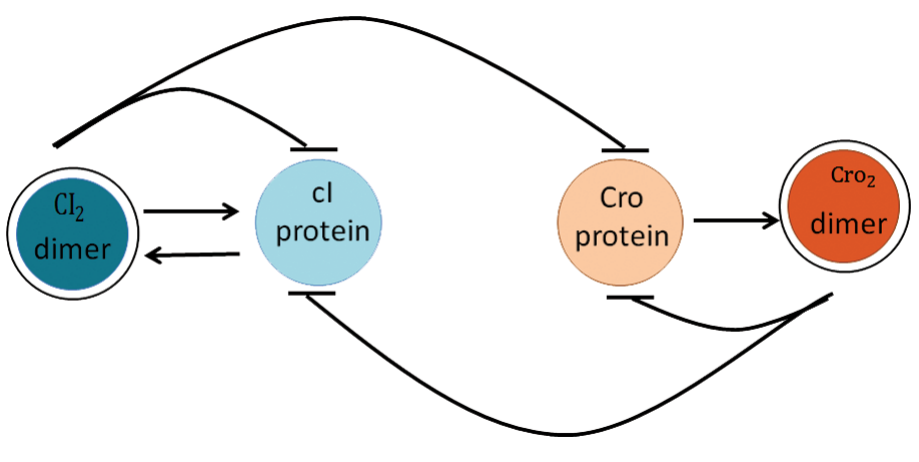
A simple bi-stt gene regulatory network for the lysislysogeny decision.

Previous studies have shown that there are a few biological factors that determine the decision between the two pathways which include number of bacteriophages inside the host bacterium, size of the host bacterium, stress, temperature and starvation (22-26, 28). One of the most well known parameters is the multiplicity of infection (MOI) which is simply the number of bacteriophages that infect the same host bacterium. The lysislysogeny decision leans towards the lysogenic pathway when a higher MOI is present (23- 25). Moreover, it has been shown that larger host bacteria increase the likelihood of the lytic pathway (22, 26). From an intracellular point of view, the expression level of two proteins cI and Cro determine the final fate of bacteriophages (Fig .1) where the former induces the lysogenic pathway and the latter induces the lytic pathway. In this regulatory network, dimer cI_2_ and protein cI have positive effects on each other but dimer cI_2_ also weakly inhibits cI. On the other hand, dimer Cro_2_ represses Cro but Cro activates dimer Cro_2_. Finally, dimers cI_2_ and Cro_2_ suppress Cro and cI respectively, as described previously by Oppenheim et al. (29).

## Materials and Methods

In this prospective study, we present a mathematical model to describe the lysis-lysogeny decision-making process where a rational player is willing to optimize longevity of its offspring. We first propose a model to measure the utility of either lytic or lysogenic actions as functions of the number of offspring that survive in their environmental circumstances. We then employ the logit-response dynamics, which is similar to the Boltzmann distribution, to find the probability of each action in a rational move.

### Poisson process

We model the infection event by the Poisson process with the average rate λ. The probability that a host bacterium, with a unit size and internal MOI λ is infected by a bacteriophage is computed as follows:

f(host bacterium MOI=μǀ average MOI=λ)=e^-λ^λ^n^/μǃ

We build this model with respect to an environment with the average MOI λ, in which each host bacterium is infected by phages.

### Lytic and lysogenic utilities

One decision is made per host bacterium during the lysis-lysogeny decision-making process. This means that all phages inside a host bacterium choose the lysis-lysogeny decision in aggregate. Thus, we can model all phages inside a host bacterium as a single decision maker. However, we do not claim that all phages inside a host bacterium behave in the same way. In specific, we do not study the mechanism by which phages inside a host bacterium reach an agreement on the final action, but we propose a mathematical model to demonstrate that the aggregated decision may be seen as a rational decision.

Given that a rational decision maker utilizes lysis and lysogeny to maximize the expected number of offspring, we define the utility of each action with the average MOI as λ in the following manner:

#### Lysogeny

In the lysogenic pathway, the phage genome is inserted into the host bacterium genome. This means that one offspring will find a host bacterium and survive, and thus the utility of lysogeny is 1 regardless of the average MOI.

#### Lysis

In the lytic pathway, the viral genome is replicated within the host bacterium, and the replicated offspring are released after killing the host bacterium. However, the released phages have no replication power, and thus start their journey for finding a new host. The utility of lysis is therefore the expected number of the released phages that find a host bacterium. Note that when many phages enter the same host bacterium, we have to divide the utility between the participants. In specific, when k phages have entered the same host bacterium, one can imagine two ways for dividing the utility of finding the host bacterium between the participants. The first method assigns 1/k to each participant, and the second one assigns the entire utility, which is 1, to the first phage that enters the bacterium. Since we do not know which phage makes the first entry, the second method assigns 1 to each phage with a probability of 1/k, the expected utility of both methods are the same. In this paper, we employ the second method and assign the entire utility to the first that enters. Assuming the phages in a bacterium have decided to lyse and release n free phages, these phages start finding an empty bacterium in the environment. In the following we compute the expected number of phages, out of those (n) phages that infect an empty bacterium.

### Balls-into-bins problem

**Fig.2:**
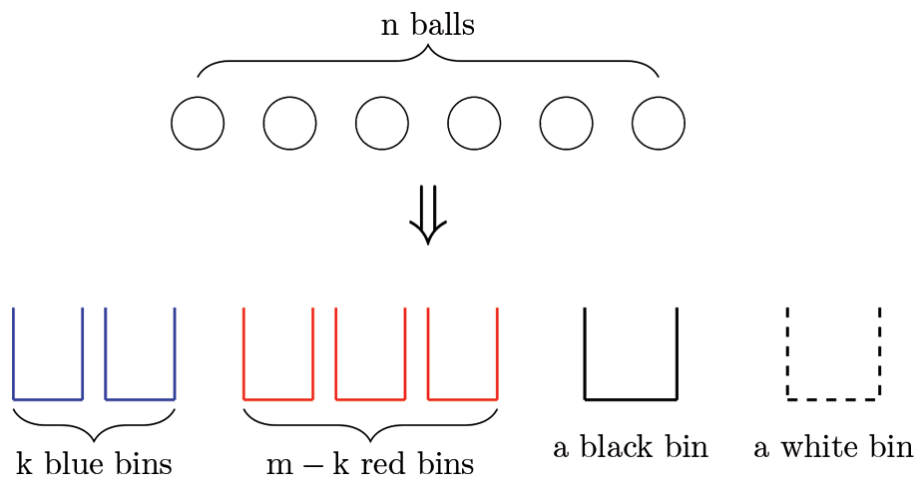
The balls-into-bins problem where we have k blue bins, m-k red bins, one black bin, and one white bin. We want to compute the expected number of balls, out of n balls that enters an empty bin. In each step we place a ball into one of the bins. The probabilities that the ball is thrown into blue, red, black, and the white bins are P_color_×k/m , P_color_×(m-k)⁄m, P_black_, and P_white_ respectively. If the ball is placed in the white bin, we pick the ball and throw it again until the ball is placed in a non-white bin.

We model the problem of phages entering an empty bacterium as a variant of balls-into-bins problem where we have n balls and four kinds of bins: k blue bins, f red bins, a black bin, and a white bin (Fig .2). Balls are representing phages and bins are representing host bacteriums. We want to compute the expected number of phages (balls), out of those (n) phages that infect an empty bacterium (blue bins). At each step, we randomly place a ball into one of these bins. In particular, the ball is thrown into the blue, red, black, and white bins with probabilities of P_color_×k/m, P_color_×(m-k)/m, P_black_ and P_white_ such that P_color_+P_black_ +P_white_=1. If the ball is placed in a nonwhite bin, we move to the next step and place the next ball into the bins. If the ball is placed in the white bin, we take the ball out and place it once more. We repeat this process until the ball is placed in a nonwhite bin and then proceed to the next ball. The goal in this stage is to count the expected number balls that has occupied blue bins. The next step is to solve the balls-into-bins problem.

### Problem solving of balls-into-bins

In this part, we propose a method to solve the corresponding balls-into-bins issue.

### Independent

Initially, we discard any kind of correlation between the balls. The bins are then randomly occupied with balls. The probability that the ball is placed in one of the blue bins is P_color_×k/m. On the other hand, the probability that the ball is placed in the white bin is P_white_ and in this situation we throw it again. Thus, the probability that the ball is placed into a blue bin, x1,k is computed by the following equation:

X_1,k_=P_color_×k/m+P_white_×X_1,k_ (2)

By rearrangement, we have:

X_1,k_=P_color_×k/m×(1-P_white_) (3)

That is the probability of a released phage that infects an empty bacterium. Thus, the expected number of phages that infect an empty bacterium can be calculated as in Equation 4.

X_n,k_=n× X_1,k_=P_color_×n×k/m×(1-P_white_) (4)

### Dependent

When a ball is placed into a blue bin, that bin is no longer empty and may not be count again. We discard this kind of dependency in the previous analysis. Here, we propose an algorithm for finding the expected number of occupied blue bins by considering dependencies. The xn,k is the expected number of occupied blue bins when we have n balls, k blue bins, and m-k red bins. Then, we have:

X_n,k_=P_color_×k/m×(1+X_n-1,k-1_)+ P_color_×m-k/m× X_n-1,k_+ P_black_× X_n-1,k_+P_white_×X_n,k_ (5)

Note that X_n,0_=X_0,k_=0 for all n≥0 and all k≥0, and one can compute X_n',k'_ for all nˊ≤n and kˊ≤m by a dynamic program (Algorithm 1).

Algorithm 1: Computing 1: for n´=0 to n do
2: x_n´,0_=0
3: end for
4: for k´=0 to m do
5: x_0,k´_=0
6: end for
7: Pˊ_color_=P_color_/(1-P_white_)
8: Pˊ_black_=P_black_/(1-_Pwhite_)
9: for n´=1 to n do
10: for k´=1 to m do
11: x_n´,k´_=P´_color_×k´/m×(1+x_n´-1,k´-1_)+P´_color_×(m-k´)/m×x_n-1,k´_+P´_black_×x_n´-1,k´_
12: end for
13: end for

### Solving the problem based on the balls-intobins issue

We first show that our problem is exactly the above variant of the balls-into-bins problem. Let P_infect_ be the probability that the phage infects a bacterium after a unit of time and let σ be the phage death rate in a unit of time. We consider each released phage as a ball and assume there are k empty bacteria out of m reachable bacteria in the environment (a bacterium is defined as reachable if the released phages infect it during their lifetime). Note that a released phage might have one of the following situations after a unit of time (e.g. an hour):

It might infect an empty bacterium with probability P_infect_×k ⁄m. We model each empty bacterium by a blue bin in the corresponding ballsinto- bins problem.

It might infect a non-empty bacterium with probability P_infect_×(m-k)⁄m. We model each nonempty bacterium by a red bin in the corresponding balls-into-bins problem.

It might die with probability (1-P_infect_)σ. We model this situation by a black bin in the corresponding balls-into-bins problem.

None of above with probability (1-P_infect_) (1- σ). We model this situation by a white bin in the corresponding balls-into-bins problem.

To compute the expected number of phages that infect an empty bacterium, which is the same as the expected number of occupied blue bins in the corresponding balls-into-bins problem, algorithm 1 is used to obtain the value of x_n´,k´_ for all 0≤n´≤n and 0≤k´≤m. X_n´,k´_ represents the expected number of empty bacteria that are infected by released phages where the number of empty bacteria is k´out of the m reachable bacteria. Having all X_n,k´_, we compute the expected number of survivors as:

$$X_{\mathrm{empty}}=\overset{m}{\underset{\mathrm{k´=0}}{\Sigma }}\mathrm{P(k´bacteria out of m are empty)}\times x_{\mathrm{n,k´}}$$

The probability that a bacterium is empty is e^-λ^ based on Equation 1, which gives:

$\mathrm{P (k´bacteria out of m are empty)=}(\overset{m}{\mathrm{k´}})e^{\mathrm{-\lambda k´}}{\left(1-e^{\mathrm{-\lambda}}\right)}^{\mathrm{m-k´}}$(7)

Therefore, the utility of the lytic pathway (i.e. the expected number of survivors) can be calculated by Equation 8.

$$X_{\mathrm{empty}}=\overset{m}{\underset{\mathrm{k´=0}}{\Sigma }}(\overset{m}{\mathrm{k´}})e^{\mathrm{-\lambda k´}}{\left(1-e^{\mathrm{-\lambda}}\right)}^{\mathrm{m-k´}}\times x_{\mathrm{n,k´}}$$

### Probability of each action

The last step is to compute the probability of each action based on their utilities. In game theory, players are assumed to act rationally and choose the action with the highest payoff (30, 31). However, the full rationality assumption is often violated in modeling human behaviors, real world situations and physics. In fact, decision makers usually deviate from a fully rational move due to many reasons such as lack of information, the required time to make decision and cognitive limitations (32-36). The noisy-best response is one of the well-known models for studying a situation when a decision is made in the real world (37-40). In this model, the probability of action i with utility ui is proportional to eβui. The noisy-best response is similar to the Boltzmann- Gibbs distribution in statistical mechanics that describe the probability distribution over various states of a system (41-43). By using the noisy-best response for modeling the lysis-lysogeny decision, one can write the probability of lysis and lysogeny actions as:

P_lysis_=e^Bulysis^/e^Bulysis^ +e^Bulysogeny^ (9) P_lysogeny_=e^Bulysogeny^/e^Bulysis^+e^Bulysogeny^ (10)

Where u_lysis_ and u_lysogeny_ represent the expected utility of the lytic and the lysogenic pathways respectively, and β can be seen as the inverse level of noise in the decision-making process. In a fully rational decision, β reaches infinity. However, in almost all real-world applications, there is a level of noise and the value of β is thus not assumed to reach infinity in these situations (In this paper we have investigated various behaviors with respect to different values of β. We have found β=1.2 to better match the behavior of bacteriophages that are reported in experimental studies, and all figures are based on β=1.2.) (37-40).

## Results

The main goal of this study was to demonstrate that the behavior of bacteriophages in a bacterium matches the behavior of a rational-decision maker, which is aimed to maximize offspring longevity.

### The utility of the lytic pathway

**Fig.3:**
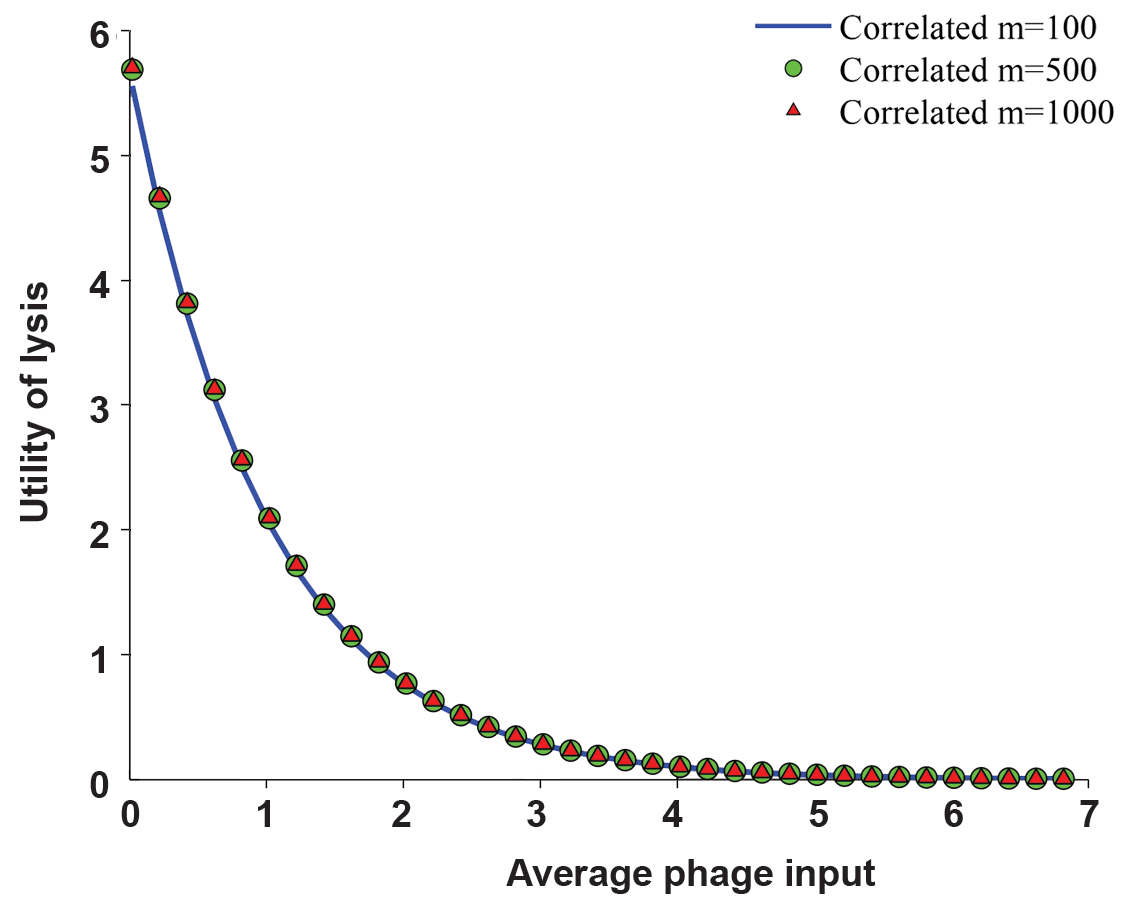
The utility of the lytic pathway vs the average multiplicity of infection (MOI). The utility of the lytic pathway for a rational decision maker in different environmental situations (i.e., different average MOIs) is given. The infection processes of released phages are independent and the infection processes of released phages are dependent. We compute the utility of the lytic pathway for m=100, m=500, and m=1000 where m is the number of reachable bacteria. The utility of the lytic pathway is not sensitive to this parameter.

For the sake of simplicity, we first computed the expected number of survivors by assuming that the infection processes of released phages are totally independent. This was done by calculating the survival probability of each released phage. Figure 3 shows the utility of the lytic pathway (i.e., the expected number of survivors) based on the average MOI, by assumption of independent infection processes. We then calculated the expected number of survivors without the independence assumption by considering totally correlated infection processes. For this, the number of reachable bacteria, m, must be known. Figure 3 shows the expected number of empty bacteria that are infected by released phages when the infection process is correlated for m of 100, 500, and 1000. The results demonstrate that the utility of the lytic pathway is almost independent of m. Moreover, the root-mean-square deviation (RMSD) between the independent case and the correlated case with m=100, m=500, and m=1000 was 0.048, 0.010, and 0.005 respectively. Given the differences were negligible, any m may be chosen for the further steps.

### The probability of the lytic pathway

**Fig.4:**
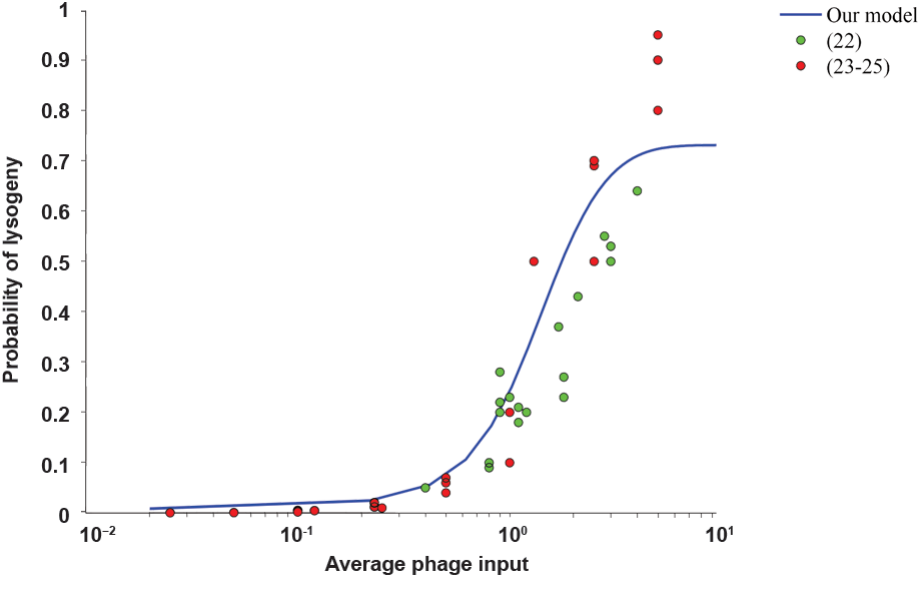
The probability of the lysogenic pathway vs. the average multiplicity of infection (MOI). The blue curve shows the prediction of our model, green circles represent the experimental data described by Zeng et al. (22), and red circles are based on experimental data described in (23-25). We compute the probability of both lytic and lysogenic pathways based on a noisy-best response dynamics. We assume the utility of lysogeny is 1 and the utility of lysis is defined as the expected number of survivors.

In the next step, we compute the probability of each action in a rational move. The probability of the lysogenic pathway is shown based on the average MOI in Figure 4 where the utility of the lysogenic pathway is set to 1 and the utility of the lytic pathway is found as in Figure 3. We use m=100, n=40, β=1, α=1.2, probability of infection=5.3e-9, density of bacteria=2.5e7 and the phage infection rate at 0.163. We compare the behavior of a rational move with the behavior of phages by using the experimental data, as previously described. In these experimental studies the probability of the lysogenic pathway is measured based on the average MOI (i.e., the average phage input). According to Figure 4, the behavior of phages could be modeled by a rational decision. We also employ the root-mean-square error (RMSE) to show the similarity between the results of the proposed model and all the experimental data together (22-25). An RMSE of 0.0910 was obtained with a range from 0.0779 based on data in (23-25) to 0.1058 based on data in (22), thus showing the accuracy of the model.

### Probability of the lytic pathway based on varying host bacterium MOI, size and concentration

The experimental data in (22) also had measured the probability of lysogeny with respect to the host bacterium MOI, size, and concentration, allowing us to examine the predictive power of our model in more detail. We consider a bacterium with size L that is infected by μ phages. Size L is the volume of the bacterium and therefore it is L times larger than the average bacterium. In this situation, a rational decision maker has to select between the lytic and the lysogenic pathways by probing the host bacterium MOI and size. The utility of each action is defined based on the average MOI rather than the host bacterium MOI and size. However, the average MOI is unknown to a rational decision maker and should be inferred based on the host bacterium and size. Given that a Poisson process models the infection process, it may be used to find the probability of having a particular average MOI by using a Bayes rule and compute the expected utility of each action by integrating over all possible average MOIs. After computing the expected utility of each action, the probability of each action is computed based on noisy-best response dynamics. We employed the same dynamics for data represented in Figure 4.

**Fig.5:**
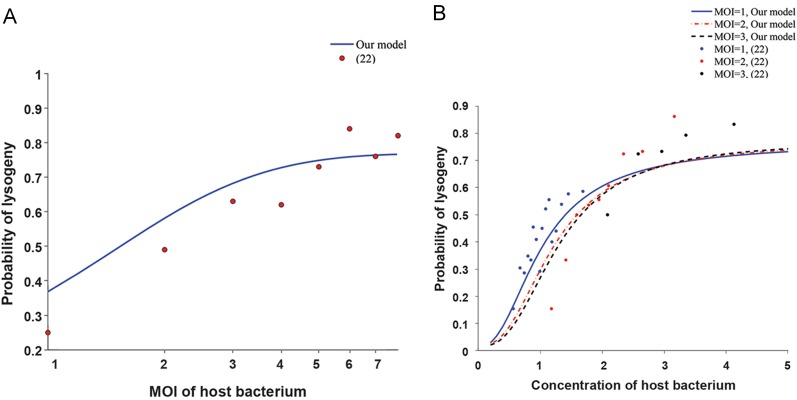
The utility of the lytic pathway based on varying host bacterium multiplicity of infection (MOI) and concentration. This figure shows that there is a clear correlation between the probability of lysogeny and the host bacterium MOI and concentration. A. The probability of choosing the lysogenic pathway based on the host bacterium MOI. The blue curve represents the behavior of a rational player based on the proposed model. Red circles represent the experimental data in (22) and B. The probability of choosing the lysogenic pathway based on the host bacterium concentration. Blue, red, and black curves represent the behavior of a rational player based on the proposed model for MOI of 1, 2 and 3 respectively. Blue, red, and black circles shows the experimental data as described by Zeng et al. (22) for MOI of 1, 2 and 3 respectively.

The probability of the lysogenic pathway based on various host MOI size and concentration is shown in Figure 5. In specific, Figure 5A represents the effect of the host bacterium MOI on the probability of the lysogenic pathway by integrating over all possible host bacterium sizes. An RMSE of 0.0763 illustrates the predictive power of the proposed model. Moreover, the probability of lysogeny at different host bacterium concentrations is shown in Figure 5B. Our model predicts a higher probability of lysogeny for a host bacterium with a lower MOI (i.e., higher size), which matches the experimental data for a fixed concentration as described by Zeng et al. (22).

## Discussion

In this study, we analyzed a fundamental phenotypic variation problem where bacteriophage lambda has to make a choice between the lytic and lysogenic pathways. Here, we present a clear quantitative model for this decision-making process. In addition, we have described the behavior of a selfish rational player, which aims to maximize its utility (i.e., the number of its offspring) in a noisy environment. We also compared this rational behavior with the observed behavior of the bacteriophage lambda previously reported (22- 25). We demonstrate that bacteriophages may be modeled as a rational player. In fact, the decision of a bacteriophage lambda can be seen as a selfish rational action maximizing the expected number of its own offspring.

## Conclusion

We present a game theoretic framework to describe a rational decision-making process in various environmental situations, which is in line with the experimentally-observed behavior of bacteriophages. Our model also confirms that a rational decision is stochastic in nature. More importantly, this study presents a clear model for demonstrating that phenotypic variation may occur when no genotypic variation exists. We believe that our model may be used as a guideline for analysis of phenotypic variation problems.
